# Autotrophic and Mixotrophic Batch Processes with *Clostridium autoethanogenum* LAbrini in Stirred Tank Bioreactors with Continuous Gassing

**DOI:** 10.3390/microorganisms14010175

**Published:** 2026-01-13

**Authors:** Anne Oppelt, Tran Yen Nhi Nguyen, Yaodan Zhang, Dirk Weuster-Botz

**Affiliations:** Chair of Biochemical Engineering, School of Engineering and Design, Technical University of Munich, Bolzmannstr. 15, 85748 Garching, Germany; anne.oppelt@tum.de (A.O.); yen-nhi.nguyen@tum.de (T.Y.N.N.); yaodan.zhang@tum.de (Y.Z.)

**Keywords:** *Clostridium autoethanogenum* LAbrini, syngas fermentation, carbon monoxide conversion, auxotrophy, D-fructose, D-xylose, L-arabinose, mixotrophy, ethanol, *meso*-2,3-butanediol

## Abstract

Simultaneous conversion of syngas and sugars is a promising approach to overcome limitations of syngas fermentation. *Clostridium autoethanogenum* LAbrini, obtained by adaptive laboratory evolution, is known to show improved autotrophic process performance. Under purely autotrophic conditions, *C. autoethanogenum* LAbrini exhibits substantially faster growth and biomass formation compared to the wild-type in fully controlled, stirred-tank bioreactors with a continuous gas supply. In mixotrophic processes, the pre-culture strategy has a significant impact on the growth and metabolic activity of *C. autoethanogenum* LAbrini. *C. autoethanogenum* LAbrini can metabolize sugars (D-fructose, D-xylose, or L-arabinose) and CO simultaneously. All mixotrophic batch processes showed increased growth and product formation compared to the autotrophic process. The mixotrophic batch process with D-fructose enabled superior production of alcohols (10.7 g L^−1^ ethanol and 3.2 g L^−1^ D-2,3-butanediol) with a heterotrophic pre-culture. Using an autotrophic pre-culture and L-arabinose resulted in a total alcohol formation of more than 13 g L^−1^. The formation of *meso*-2,3-butanediol (>0.50 g L^−1^) occurred exclusively under mixotrophic conditions. Thus, *C. autoethanogenum* LAbrini, clearly representing notable improvements over the wild-type strain in mixotrophic batch processes, offers a good basis for further strain improvements to shift the product range even further towards more reduced products.

## 1. Introduction

Syngas fermentation is a promising biotechnological approach involving anaerobic microorganisms to convert C1 gases into value-added products, such as platform chemicals or liquid fuels [1,2,3]. Syngas primarily consists of nitrogen (N_2_), carbon monoxide (CO), hydrogen (H_2_), and carbon dioxide (CO_2_) and can be obtained from the gasification of energy-rich and renewable biomass, such as straw, sugar cane bagasse, green waste, or rice husks [1,4]. However, the composition of the syngas depends on the feedstock, operation strategy, and oxidizing agent [5,6,7]. In a study, using oxygen and steam as gasifying agents, the entrained-flow gasification of torrefied wood produces a gas mixture containing 45.0% CO, 25.6% CO_2_, and 27.9% H_2_, as well as trace amounts of N_2_, methane (CH_4_), and ammonia (NH_3_) [8]. By contrast, fluidized-bed gasification of cypress sawdust using air yields a composition containing 6.9% CO, 18.1% CO_2_, 5.6% H_2_, 68.0% N_2_, and 1.4% CH_4_ [9].

Autotrophic, acetogenic anaerobes, including various species of the family *Clostridiaceae*, can metabolize either CO and/or CO_2_ together with H_2_ through the Wood-Ljungdahl pathway (WLP) [10,11,12]. The WLP is the most energy-efficient naturally occurring CO_2_-fixation pathway [10]. In the WLP, the key enzyme, acetyl-CoA, is generated through several reduction reactions [1,13]. Among others, CO dehydrogenase/Acetyl coenzyme A (Acetyl-CoA) synthase couples its two parallel branches enzymatically by catalyzing CO, a methyl group, and a coenzyme A to acetyl-CoA [1,13,14]. For this purpose, either H_2_ or CO can supply the required electrons, although the latter is the preferred thermodynamic electron donor [13]. Acetyl-CoA is indispensable for anabolism and product formation [13]. The final product spectrum largely depends on the *Clostridia* species involved. For instance, the closely related species *Clostridium autoethanogenum*, *C. coskatii*, *C. ljungdahlii*, and *C. ragsdalei* produce acetate, ethanol, and 2,3-butanediol, whereas *C. carboxidivorans* builds acetate, ethanol, butyrate, and 1-butanol [1,14]. By contrast, *C. muellerianum* forms caproate and 1-hexanol as well as acetate [15].

Still, one of the biggest challenges in anaerobic syngas fermentation is the poor solubility of CO and H_2_ in aqueous solutions, which limits gas-liquid mass transfer and results in low product concentrations [16,17]. However, acetogens demonstrate high adaptability to various syngas compositions, exhibit notable tolerance to syngas impurities, and are already used industrially [1,18,19]. For instance, Synata Bio is currently working on a pilot-scale process using *C. ljungdahlii* [20]. LanzaTech is the most prominent example, employing engineered *C. autoethanogenum* for large-scale applications to produce ethanol, acetone, and isopropanol [14,21].

Beyond the autotrophic metabolism using the WLP, many *Clostridia* spp. are also able to utilize carbohydrates [22]. For instance, *C. autoethanogenum* JA1-1 can grow with D-fructose or pentoses, such as L-arabinose, D-ribose, and D-xylose [22,23]. As has been shown for *C. ljungdahlii*, D-fructose is taken up via fructose-specific phosphotransferase systems and converted to D-fructose-1,6-bisphosphate before entering the Embden–Meyerhof–Parnas (EMP) pathway [24]. Upon uptake into the cell, L-arabinose or D-xylose is converted into D-xylose phosphate or L-ribose phosphate, respectively. These phosphorylated sugars then enter the pentose phosphate pathway (PPP), where they undergo several intermediate steps. The formed intermediates are then assimilated into EMP [25,26,27]. Then acetyl-CoA is produced by the pyruvate:ferredoxin oxidoreductase (PFOR), which releases CO_2_ and reduces ferredoxin [26].

During sugar catabolism, CO_2_ is primarily lost through the decarboxylation reaction described above [26,28]. Such CO_2_ loss is unavoidable when the resulting product or metabolite is more reduced than the substrates [28]. To meet the required electron demand for CO_2_ fixation, additional electron donors, such as CO or H_2_, can be supplied. In syngas fermentation, this strategy is known as anaerobic, nonphotosynthetic (ANP) mixotrophy [28,29]. However, challenges remain, particularly regarding the stoichiometric electron requirements for CO_2_ fixation and carbon catabolite repression (CCR) [29].

In a fed-batch process with *C. autoethanogenum* JA1-1 in a 7 L-STR, feeding of D-xylose and CO resulted in a higher conversion rate of CO and improved productivity compared to a batch process where first D-xylose and then CO was supplied [30]. Abubackar et al. [31] have shown in a continuous STR process that this strain has a preferred uptake of D-xylose over CO. Furthermore, Jones et al. [32] had demonstrated that *C. autoethanogenum* JA1-1 can utilize D-fructose and syngas simultaneously in anaerobic flasks. Moreover, in our first study, we observed that the simultaneous supply of syngas (artificial syngas mixture: 39% N_2_, 30% CO, 22% H_2_, 9% CO_2_) and pentoses, particularly L-arabinose and D-xylose, enhances growth and product formation in a fully controlled 1 L-STR with *C. autoethanogenum* JA1-1. The mixotrophic processes yield higher alcohol-to-acid ratios than purely autotrophic batch processes. Notably, co-feeding L-arabinose and syngas increases not only biomass but also enables *meso*-2,3-butanediol production, a phenotype not observed under strictly heterotrophic conditions. However, no simultaneous utilization of syngas and D-fructose was observed [33].

Mostly, acetate is the primary and sole product of many acetogens, and is typically produced in higher yields in contrast to parallel-formed products; however, its low economic value limits its commercial attractiveness [34]. *Acetobacterium woodii* produces solely acetate, achieving a final acetate space-time yield of 148 g L^−1^ d^−1^ in a continuously operated STR equipped with a submerged microfiltration membrane [35]. Therefore, metabolic engineering efforts often aim to reduce or eliminate acetate formation to increase yields of higher-value products, such as acetone, 2,3-butanediol, 1-butanol, ethanol, or lactate, as well as to implement novel product routes [1,14]. This is feasible to some extent, as the WLP and the energy conservation of acetogens have already been extensively studied [2,10,13,14,26,34,36,37].

Although many genomes of acetogens have been published, a substantial proportion of their genes remain unannotated and, thus, their functions remain unresolved [14,38]. Consequently, the understanding of genotype-phenotype relationships remains very limited, which makes targeted metabolic engineering challenging [39,40,41]. A promising approach is adaptive laboratory evolution (ALE) [37]. In contrast to targeted metabolic engineering, ALE leverages evolutionary processes to enable phenotypic adaptation, thereby uncovering previously unknown regulatory mechanisms [37,38].

In the study by Ingelman et al. [42], three different ALE strategies were employed to improve the wild-type strain of *C. autoethanogenum* JA1-1. Seven enhanced strains were isolated from the ALE experiments. Genome sequencing of the evolved strains revealed 25 mutations, including two genomic regions with recurrent mutations. The isolate “LAbrini” stood out in particular due to its superior growth rate and product profile. Reverse genetic engineering in sporulation-related genes was able to reproduce the advantageous trait of “LAbrini”. The study develops the novel *C. autoethanogenum* LAbrini strain, which is expected to enable faster growth, independence from complex nutrients, and reduced acetate formation [38,43].

As aforementioned, *C. autoethanogenum* LAbrini should be able to perform independently of complex nutrients, especially yeast extract. Yeast extract is known to contain amino acids, ribonucleotides, minerals, vitamins, and peptides [39]. However, its exact composition varies depending on the yeast raw material and the production process [39]. It has been demonstrated that a large number of acetogens showed no microbial activity with syngas unless yeast extract was added [14]. Also, *C. autoethanogenum* JA1-1 exhibited no growth or product formation during anaerobic cultivation in serum bottles containing 25 mL medium (syngas mixture: 20% CO, 20% CO_2_, 10% H_2_, 50% N_2_) [40]. However, eliminating yeast extract from fermentation media has several advantages, e.g., experimental reproducibility, simplifying metabolic modelling, and minimizing operating costs on an industrial scale [37].

*C. autoethanogenum* LAbrini is highly relevant for laboratory studies and industrial applications. However, as this engineered strain became commercially available in 2024, reaction engineering studies are currently limited to the work of Ingelman et al. [43]. To date, no batch processes in STR with fully controlled reaction conditions, including product quantification and gas consumption analysis, have been reported for *C. autoethanogenum* LAbrini. Furthermore, there is a lack of understanding regarding the potential impact of the pre-culture strategy on mixotrophic performance in acetogens. And it has been suggested that ANP mixotrophy may counteract CO_2_ loss during the decarboxylation of pyruvate to acetyl-CoA under heterotrophic conditions. This makes mixotrophic fermentation an increasingly important area of research in syngas fermentation.

First, in this work, we compare batch processes of *C. autoethanogenum* LAbrini with and without yeast extract, relating these results to our earlier findings with the wild-type *C. autoethanogenum* JA1-1 [33]. We then present three mixotrophic batch processes using D-fructose, employing three different pre-culture preparations to check for variations in process performance. Finally, we will compare mixotrophic batch processes involving either L-arabinose, D-fructose, or D-xylose. We will show that *C. autoethanogenum* LAbrini metabolizes CO and the provided sugars simultaneously, thereby substantially increasing the growth rate, gas uptake rate, product formation, and the alcohol-to-acid ratio compared to the autotrophic reference batch process. In addition, we will show that *C. autoethanogenum* LAbrini also produces *meso*-2,3-butanediol when utilizing CO and L-arabinose simultaneously.

## 2. Materials and Methods

### 2.1. Media, Microorganism, and Cryo-Conservation

The preparation, anaerobization, and sterilization of media for pre-cultures, as well as for bioreactor fermentations, were adapted from Doll et al. [41]. Its detailed composition is listed in the Supporting Information (Table S1). For the pre-cultivation, 15 g L^−1^ 2-(N-morpholino)ethanesulfonic acid (MES) was added to the medium as a buffer, along with 1 g L^−1^ yeast extract. To anaerobize the medium, it was boiled for 20 min and then purged with N_2_ for at least 30 min. Under anaerobic conditions, 100 mL of medium was aliquoted into either 250 mL or 500 mL anaerobic flasks. The medium was then autoclaved.

An active culture of *C. autoethanogenum* LAbrini (DSM 115981) was obtained from the German Collection of Microorganisms and Cell Cultures (DSMZ, Braunschweig, Germany). For the cryo-conservation, exclusively medium in 250 mL anaerobic flasks were used. For a master cell bank (MCB), 5 mL of the active culture, supplemented with 0.4 g L^−1^ cysteine hydrochloride as reducing agent and 10 g L^−1^ D-fructose, was cultivated in sterile anaerobic 100 mL medium. The anaerobic flask was incubated at 37 °C with a shaking frequency of 100 min^−1^ until an optical density at 600 nm (OD_600_) of approximately 0.9 was reached. In a second cultivation step, 10 mL of the cell suspension was transferred to 100 mL medium containing 0.4 g L^−1^ L-cysteine hydrochloride and 12 g L^−1^ D-fructose. The anaerobic flask was incubated as before until an OD_600_ of approximately 0.9 was achieved. The MCB was supplemented with 5% (*v*/*v*) dimethyl sulfoxide (DMSO), aliquoted, and transferred to sterile, anaerobic Hungate tubes (16 × 125 nm, Dunn Labortechnik, Asbach, Germany) and stored at −80 °C.

For the preparation of the working cell bank (WCB), 6 mL of a MCB culture was added to 100 mL anaerobic medium containing 0.4 g L^−1^ L-cysteine hydrochloride and 10 g L^−1^ D-fructose. The anaerobic flask was incubated as before at 37 °C and 100 min^−1^ until an OD_600_ of approx. 0.9 was reached. The WCB culture was supplemented with 5% (*v*/*v*) DMSO, aliquoted, and transferred to sterile, anaerobic Hungate tubes and stored at −80 °C.

### 2.2. Autotrophic, Heterotrophic, and Mixotrophic Pre-Cultures

For preparing the autotrophic pre-cultures, exclusively 100 mL medium in 500 mL anaerobic flasks were used. The 500 mL anaerobic flasks were exposed to a gas mixture of 0.70 bar_abs_ N_2_, 0.54 bar_abs_ CO, 0.40 bar_abs_ H_2_, and 0.16 bar_abs_ CO_2_ at 2 bar_abs_ as an autotrophic substrate supply before inoculation. The composition of the artificial syngas used was adapted from Rückel et al. [42], who derived it from the gasification of torrefied wood. For the first autotrophic pre-culture stage, 5 mL of WCB culture was incubated in anaerobic medium containing the autotrophic substrate as well as additional 0.4 g L^−1^ L-cysteine hydrochloride, at 37 °C for at least 60 h at 100 min^−1^. For the second autotrophic pre-culture stage, 10 mL of the inoculum from the first pre-culture after incubation was added to an anaerobic flask containing the autotrophic substrate and 0.4 g L^−1^ of L-cysteine hydrochloride.

For preparing the heterotrophic pre-cultures, exclusively 100 mL medium in 250 mL anaerobic flasks (1 bar_abs_ N_2_) were used. For the first heterotrophic pre-culture stage, 2.5 mL of WCB culture and 5 g L^−1^ D-fructose was incubated in 100 mL anaerobic medium at 37 °C and 100 min^−1^ for 60 h. The second heterotrophic pre-culture stage consisted of a 10 mL inoculum from the first pre-culture after incubation. Additionally, 5 g L^−1^ D-fructose and 0.4 g L^−1^ of L-cysteine hydrochloride was added to the anaerobic medium.

The same procedure as for the anaerobic pre-cultures was used for mixotrophic pre-cultures. For the first mixotrophic pre-culture stage, 5 mL of a WCB culture and 10 g L^−1^ D-fructose as a heterotrophic carbon source were incubated in 100 mL medium in 500 mL anaerobic flasks containing the autotrophic substrate for 60 h at 37 °C and 100 min^−1^. In a second pre-culture stage, 12.5 g L^−1^ D-fructose and 0.4 g L^−1^ of L-cysteine hydrochloride were added to 100 mL medium, along with 10 mL inoculum after incubation from the first stage.

For all pre-cultivation strategies, at the end of the exponential growth phase of the second pre-culture stage, *C. autoethanogenum* LAbrini was harvested by centrifugation (10 min, 3620 rcf, Rotica 50 RS, Hettich GmbH & Co. KG, Tuttlingen, Germany) under anaerobic conditions. The cell pellets were resuspended in 10 mL anaerobic phosphate-buffered saline (12 mM phosphate). A syringe with an injection needle was used to draw up the cell suspension and inject it via a lid port of the stirred-tank bioreactor equipped with a septum. For all batch processes, the stirred-tank bioreactor was inoculated at an initial CDW concentration between 0.03 and 0.04 g L^−1^.

### 2.3. Setup of Continuously Gassed Stainless Steel, Stirred-Tank Bioreactor

All batch processes were carried out in a fully controlled, stainless steel, 2.4-L stirred-tank bioreactor (STR) (KLF2000, Bio-Engineering, Wald, Switzerland) with a working volume of 1 L (V_total_ = 2.4 L) at 1 bar_abs_ [33]. The STR was stirred with two Rushton turbines at 1200 rpm (volumetric power input of 15.1 W L^−1^) and was continuously gassed with 5 NL h^−1^ (0.083 vvm) artificial synthesis gas. The power input was selected to maximize gas-liquid mass transfer while minimizing excessive shear effects on the cells and maintaining low mechanical stress on the reactor. The pH was controlled at a set-point of pH 6.0 using 3 M sodium hydroxide (NaOH) or 0.5 M sulfuric acid (H_2_SO_4_). The pH was measured using a sterilized pH sensor (405-DPAS-SC-K8S/120, Mettler Toledo, Germany). The redox potential was not controlled, but recorded using a sterilizable redox sensor (Pt4805-DPAS-SC-K8S/120, Mettler Toledo, Germany). The temperature was controlled at 37 °C. For safety purposes, a pressure probe and a mechanical safety valve that opens at 3.5 bar_abs_ were installed.

The gas mixing system consisted of four thermal mass flow controllers (P-702CV-6K0R-RAD-33-V, Bronkhorst, Reinach, Switzerland). An artificial syngas composition was established by adjusting the flow rates of the individual gases to 1.95 NL h^−1^ N_2_ (p_N2_ = 0.39 bar_abs_; 39.4 (*v*/*v*)), 1.50 NL h^−1^ CO (p_CO_ = 0.30 bar_abs_; 29.8 (*v*/*v*)), 1.10 NL h^−1^ H_2_ (p_H2_ = 0.22 bar_abs_; 22.0 (*v*/*v*)), and 0.45 NL h^−1^ (p_H2_ = 0.09 bar_abs_; 8.9 (*v*/*v*)) [33,42].

The STR, together with 1 L medium, was sterilized in situ at 121 °C for 21 min. To achieve yeast-free cultivation conditions, yeast extract (YE) was excluded from the medium composition. 10 mL vitamin stock solution was added aseptically after the STR was cooled down to 37 °C. The medium was then anaerobized by gassing with sterile 20 NL h^−1^ N_2_ for at least 1 h. The composition of the artificial syngas (5 NL h^−1^) was then adjusted for at least 16 h before inoculation to ensure fully saturation of the medium with CO and H_2_. For mixotrophic processes, sterile, anaerobic stock solutions of either 250 g L^−1^ D-fructose, 250 g L^−1^ D-xylose, or 250 g L^−1^ L-arabinose were prepared. To achieve an initial sugar concentration between 16–19 g L^−1^, the appropriate volume of the respective sugar stock solution was aseptically injected through a septum. Immediately before inoculation, 0.4 g L^−1^ of sterile, anaerobic L-cysteine hydrochloride was added as a reducing agent to ensure a redox potential of less than −200 mV.

Autotrophic batch experiments were conducted in two individual duplicates, while the mixotrophic batch processes were each conducted once.

### 2.4. Analytical Methods

Samples of the fermentation broth were withdrawn with a syringe with injection needle through a septum fixed at a side port of the STR to determine biomass and product concentrations.

At the end of an autotrophic batch process, 3 × 45 mL cell suspension was harvested and centrifuged for 20 min at 3620 rcf (Rotica 50 RS, Hettich GmbH & Co. KG, Tuttlingen, Germany) under anaerobic conditions. The liquid supernatant was discarded, and the cell pellet was dried at 80 °C for 48 h to measure the CDW. The cell dry weight (CDW) concentrations of samples from all batch processes were estimated by OD_600_ measurements based on the previously determined correlation factor of 0.40 g L^−1^ × OD_600_. A sigmoidal function was applied to estimate the growth curve based on the measured CDW concentrations [44]. The GRG non-linear algorithm of the Microsoft Excel Solver plug-in was used to minimize the sum of squares error. The exponential batch-specific growth rate (µ_expt_) was calculated using non-linear regression analysis of at least three estimated CDW concentrations from the exponential growth phase.

A HPLC (LC-2030C, Shimadzu, Kyoto, Japan) with a cation exchange column (Aminex HPX-87H, Bio-Rad, Munich, Germany) and a refractive index detector (RID-20A, Shimadzu, Kyoto, Japan) was used to analyse the sugar and the product concentrations (organic acids and alcohols). Therefore, samples with 1 mL of the fermentation broth were sterile-filtrated into vials prior to measurement. All analyses were conducted isocratically using 5 mM H_2_SO_4_ at a flow rate of 0.6 mL min^−1^ and a column temperature of 60 °C.

To determine the gas uptake and production rates, the peak areas of N_2_, CO, H_2_, and CO_2_ in the reactors’ exhaust gas were recorded for every 12 min using a micro gas chromatography system (Micro GC 490, Agilent Technologies Inc., Santa Clara, CA, USA). The volumetric flow rates of the exhaust gas were continuously measured using a thermal mass flow meter (F-101D-RAD-33-V, Bronkhorst, Reinach, Switzerland). The peak areas of the individual gases were converted into their respective partial pressures. Since the thermal mass flow meter was calibrated on N_2_, a discrepancy arises between the volumetric flow rates indicated by the device and the actual volumetric flow rates. Therefore, a gas conversion factor was used to calculate the actual volumetric flow rates of the individual gases. The actual volumetric flow rates were then multiplied by the corresponding partial pressures and converted into the gas uptake and production rates in [mmol L^−1^ h^−1^]. The total CO consumption and CO_2_ production were determined by integrating the rates over the entire process time.

For the carbon balance, the total produced carbon in mmol C L^−1^ (biomass, ethanol, acetate, D-2,3-butanediol, *meso*-2,3-butanediol, and CO_2_) was divided by the total consumed carbon in mmol C L^−1^ (CO, yeast extract, and the respective heterotrophic sugar).

## 3. Results and Discussion

### 3.1. Autotrophic Batch Processes with C. autoethanogenum LAbrini

#### 3.1.1. Comparison of *C. autoethanogenum* LAbrini with the Wild-Type Strain

**Figure 1:**
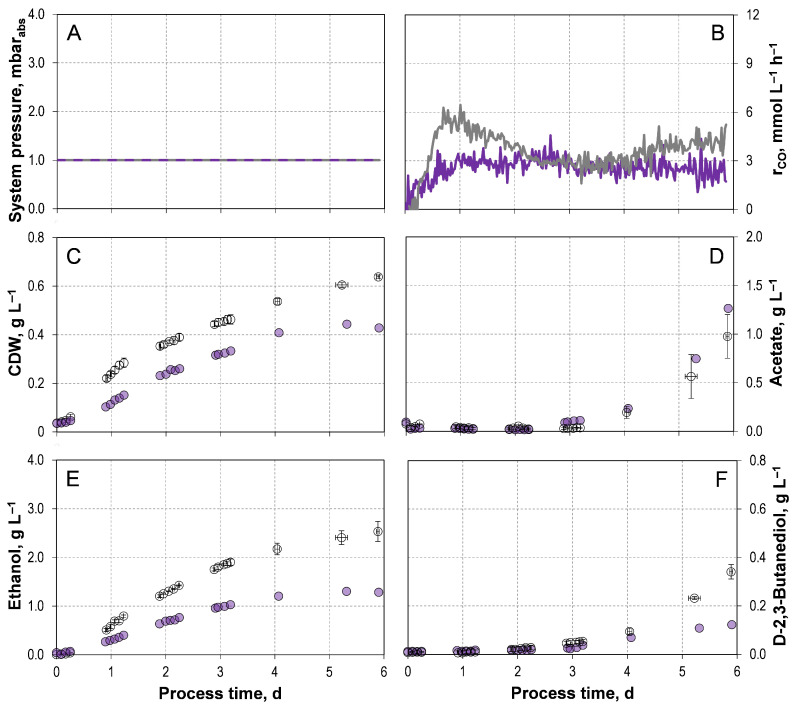
Autotrophic batch processes of *C. autoethanogenum* LAbrini with 1 g L^−1^ yeast extract (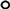) and without yeast extract (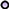) in stirred-tank bioreactors with continuous gassing (artificial gas mixture of N_2_, CO, CO_2_, and H_2_ in a ratio of 39:30:22:9). (F_gas_ = 5 NL h^−1^, 37 °C, pH 6.0, and P V^−1^ = 15.1 W L^−1^). (**A**) System pressure; (**B**) Carbon monoxide uptake rate; (**C**) Cell dry weight concentration (CDW); (**D**) Acetate concentration; (**E**) Ethanol concentration; (**F**) D-2,3-butanediol concentration. The error bars indicate the minimum and maximum values of two individual autotrophic batch processes.

The corresponding process data from two independent autotrophic batch processes with *C. autoethanogenum* LAbrini supplemented with 1 g L^−1^ yeast extract (YE) are shown in Figure 1. A final CDW concentration after six days of 0.63–0.65 g L^−1^ was reached. The final acetate concentrations ranged from 0.75–1.20 g L^−1^. The final and maximal alcohol concentrations were 2.33–2.74 g L^−1^ ethanol and 0.31–0.37 g L^−1^ D-2,3-butanediol, respectively. The carbon balance was closed to 96.44–99.82%. The performance data are summarized in Table 1 in comparison to the data of *C. autoethanogenum* JA1-1 [33], as the reactor geometry, media composition, and process conditions are identical to those from our previous study with the wild-type strain *C. autoethanogenum* JA1-1.

All product concentrations and, consequently, the alcohol-to-acid ratios remain similar between the two strains. However, *C. autoethanogenum* LAbrini exhibits increased carbon assimilation, resulting in a final CDW concentration that is 20% higher compared to the wild-type strain. Also, the exponential batch-specific growth rate µ_exp_ of 0.08–0.09 h^−1^ is improved by 30% compared to the wild-type strain. Furthermore, *C. autoethanogenum* LAbrini enters the exponential growth phase after 4–6 h, whereas the wild-type strain exhibits a pronounced *lag* phase of approximately one day [33].

In the batch process with *C. autoethanogenum* JA1-1, acetate accumulation occurs during the initial 1.5 days of process and is followed by a decrease [33]. This indicates its ferredoxin-dependent reduction from acetate to ethanol [1,33]. This pattern is not observed with *C. autoethanogenum* LAbrini. The absence of early acetate formation suggested that the adenosine triphosphate (ATP) is not provided through the acetate-dependent substrate-level phosphorylation. Consequently, the ATP required for the WLP must be supplied via the membrane-bound ATPase driven by the Rnf-complex. This ATP provision appears sufficient to maintain autotrophic metabolism at this stage of the process.

Ingelman et al. [42] identified mutations in the genome of *C. autoethanogenum* LAbrini that are located in two key genetic loci. These mutations may explain the phenotypic differences observed between the wild-type strain and its derivative. A point mutation (cytosine to adenine) was identified in gene CLAU_3129 (spo0A), which encodes the sporulation transcriptional activator Spo0A [43]. Spo0A is known as a key regulator of sporulation and solventogenesis in *Clostridia* [45]. Through this change, the derivative exhibits faster growth than the wild-type strain [43]. A 2717-bp deletion occurred in the genes CLAU_3832-3830. These genes are involved in the metabolism of pyrimidines. Through faster RNA monomer synthesis, potentially increasing translation rates and thus accelerating growth [43].

However, it is worth noting that for the autotrophic batch process with *C. autoethanogenum* LAbrini, a two-stage autotrophic pre-culture was employed. In contrast, for the batch process with the wild-type, it was a one-stage heterotrophic pre-culture [33]. A batch process in the STR with *C. autoethanogenum* LAbrini after a heterotrophic pre-culture exhibited no growth over two days .

#### 3.1.2. Comparison of Autotrophic Batch Processes with and Without Yeast Extract

The autotrophic batch process with *C. autoethanogenum* LAbrini without YE in the medium showed clearly reduced biomass and product formation compared to the wild-type (Figure 1), with the exception of acetate formation. Furthermore, in the absence of YE, the carbon content in biomass decreased by 45% (Table 1). The final concentrations of ethanol (1.30 g L^−1^) and D-2,3-butanediol (0.12 g L^−1^) were 1.95 and 2.83 times lower than in the batch process supplemented with YE. Consequently, the alcohol-to-acetate ratio was lower in the batch process without YE, reaching 1.12 g g^−1^.

The unexpected reduced process performance without YE might indicate a still persistent limitation of essential nutrients. Although defined amounts of minerals, vitamins and trace elements were supplemented to the medium, certain components may still have been insufficient, as YE also provides undefined quantities of these critical nutrients [39]. Nevertheless, *C. autoethanogenum* JA1-1 exhibits no metabolic activity in the absence of YE [40], *C. autoethanogenum* LAbrini clearly demonstrated growth and product formation under yeast-free conditions.

Comparing exponential batch-specific growth rates (µ_exp_ = 0.06 h^−1^) for *C. autoethanogenum* LAbrini without YE to those of the wild-type strain with 1 g L^−1^ YE, it becomes evident that they are essentially identical (Table 1). The same applies to their biomass profiles as a function of process time, except for the final CDW concentration [33]. This is in contrast to the data reported by Ingelman et al. [43]. They reported maximum specific growth rates of 0.08 h^−1^ with *C. autoethanogenum* LAbrini without YE and µ_max_ = 0.02 h^−1^ with the wild-type strain with YE. However, these studies were performed in uncontrolled anaerobic flasks with another initial gas composition (50% CO, 20% H_2_, 20% CO_2_, and 10% Argon) at a reduced initial pH 5.

For our following studies, we decided to keep the YE concentration of 1 g L^−1^, as the autotrophic process with YE resulted in higher final CDW concentrations and a more favorable alcohol-to-acetate ratio. Moreover, using YE facilitates direct comparability with existing literature on *C. autoethanogenum* JA1-1.

### 3.2. Mixotrophic Batch Processes with C. autoethanogenum LAbrini

#### 3.2.1. Comparison of Varying Pre-Culture Preparations for Mixotrophic Batch Processes with D-Fructose

**Figure 2:**
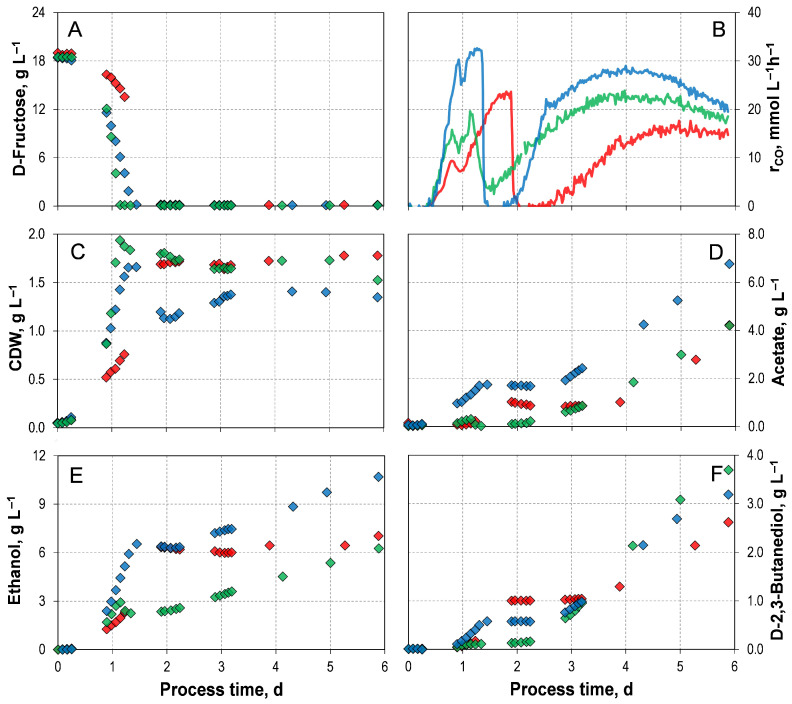
Mixotrophic batch processes of *C. autoethanogenum* LAbrini with D-Fructose using autotrophic pre-cultures (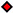), mixotrophic pre-cultures (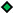), and heterotrophic pre-cultures (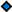) in stirred-tank bioreactors with continuous gassing (390 mbar N_2_, 300 mbar CO, 220 mbar H_2_, and 90 mbar CO_2_). (F_gas_ = 5 NL h^−1^, 1.0 bar_abs_, 37 °C, pH 6.0, and P V^−1^ = 15.1 W L^−1^). (**A**) D-Fructose concentration; (**B**) Carbon monoxide uptake rate; (**C**) Cell dry weight concentration (CDW); (**D**) Acetate concentration; (**E**) Ethanol concentration; (**F**) D-2,3-butanediol concentration.

Three mixotrophic batch processes were conducted in STRs with continuous gassing, each using an initial D-fructose concentration between 18.5–19.0 g L^−1^. D-Fructose was selected as the heterotrophic carbon source, as the active culture obtained from DSMZ had been prepared with D-fructose. The mixotrophic batch processes differed in the pre-culture strategy and were inoculated with either autotrophic, mixotrophic, or heterotrophic pre-cultures. The corresponding process performance data are shown in Figure 2 and Table 2.

Simultaneous utilization of D-fructose and CO occurred in all three mixotrophic batch processes (Figure 2A,B). Once D-fructose was completely consumed, the maximum CO uptake rate was reached. After its maximum, CO consumption abruptly dropped. And after a short period of no CO uptake, increasing CO consumption was observed until the end of the process. In the mixotrophic batch process with a mixotrophic pre-culture, the fastest consumption of the sugar was observed at the lowest maximum CO uptake rate of 23.88 mmol L h^−1^. The batch process with heterotrophic pre-culture showed the highest overall CO consumption of 2644.26 mmol L^−1^ and the highest maximal CO uptake rate of 32.64 mmol L^−1^ h^−1^. No H_2_ uptake was observed in any of the processes (Supporting Information; Figure S3).

As anticipated, not only CO uptake but also biomass and product formation were increased with the addition of D-fructose compared to the autotrophic reference batch process (with YE) (Table 2). In general, once D-fructose was depleted, and the maximum CO uptake rate was reached, the CDW concentration also approached its maximum (Figure 2C). Then, the CDW concentration remained almost stable, except in the process with heterotrophic pre-cultures. Here, the biomass concentration declined briefly before recovering and stabilizing from day 3 onwards. However, in the mixotrophic process inoculated with mixotrophic pre-culture, a CDW concentration of 1.94 g L^−1^ was achieved after 26 h, representing the fastest biomass accumulation. Compared to the autotrophic reference batch process, this corresponds to an approximately 200% increase. Furthermore, the exponential batch-specific growth rates exceeded those of the autotrophic reference batch process, which is a 200% improvement.

In all processes, acetate, ethanol, and D-2,3-butanediol production started synchronously after a short *lag* phase (Figure 2D–F). Once D-fructose was fully depleted, CO uptake temporarily collapsed, and the CDW concentration reached its maximum; each product then experienced a local plateau. The plateau was less pronounced for acetate and ethanol in the process with the heterotrophic pre-culture. In other words, as no heterotrophic or autotrophic substrates were consumed, no carbon can be metabolized, and the product formation by the cells stopped. Once CO uptake resumed, the formation of all products continued accordingly. Although the mixotrophic process with a heterotrophic pre-culture yielded the highest final acetate concentration (6.77 g L^−1^), it also produced the highest ethanol concentration of 10.69 g L^−1^.

*C. autoethanogeum* LAbrini clearly demonstrates that the choice of pre-culture strategy has a significant impact on mixotrophic fermentations using syngas and D-fructose as substrates. While the overall trends in substrate consumption, growth, and product formation are broadly similar across the shown processes, substantial differences arise in the absolute CO consumption and the particular final product concentrations. A study of *C. ljungdahlii* investigated the influence of heterotrophic, mixotrophic, and autotrophic pre-cultures on a mixotrophic batch process in a 250 mL-STR [46]. They used 2.5 g L^−1^D-fructose as well as a syngas mixture consisting of 50% N_2_, 20% CO, 20% CO_2_, and 10% H_2_. Growth and the formation of acetate and ethanol were independent of the pre-culture used [46].

*C. autoethanogenum* LAbrini can utilize D-fructose and CO simultaneously. This proves that the EMP and WLP are operating in parallel. The activity of both metabolic pathways evidenced no carbon catabolite repression (CCR), which is a common challenge in mixotrophic processes [29,31,33]. In our previous study using the wild-type strain *C. autoethanogenum* JA1-1 with solely a heterotrophic pre-culture strategy, an opposite behavior was observed: no simultaneous utilisation of CO and D-fructose (16.4 g L^−1^), and thus CCR [33]. The heterotrophic pre-culture was prepared with D-fructose. However, it is worth noting that *C. autoethanogenum* JA1-1 was obtained as a freeze-dried culture and cryopreserved according to the DSMZ protocol, utilizing D-xylose as the carbon source [33]. As the process conditions for these processes with the heterotrophic pre-cultures were identical, the phenotypes of *C. autoethanogenum* JA1-1 and its mutant, *C. autoethanogenum* LAbrini, can be compared directly. As a result of the simultaneous uptake of both carbon sources, the derivative fixed 88% more carbon into CDW. In terms of the product spectrum, the wild-type strain produced less unfavorable acetate (3.78 g L^−1^) [33]. However, the more favored alcohol formation was markedly enhanced with the mutant. In the process involving the heterotrophic pre-culture, the total alcohol concentration reached 13.88 g L^−1^. In contrast, the wild-type strain produced only 4.44 g L^−1^ of ethanol and did not form the longer-chain alcohol D-2,3-butanediol [33]. In this respect, the mutant strain is clearly superior to the wild-type.

Interestingly, the formation of all products exhibits a biphasic pattern: while D-fructose is available, biomass and product synthesis proceed in parallel; after sugar depletion, product formation becomes uncoupled from growth. It was expected that cells would become partially inactive or lyse due to insufficient energetic support for the WLP in the second phase, as the EMP no longer supplied further ATP and reduction equivalents. *C. autoethanogenum* LAbrini did not experience a significant decrease in CDW concentration. Acetate, ethanol, and D-2,3-butanediol formation continued until the process ended. In contrast, *C. autoethanogenum* JA1-1 exhibited a biomass declined after D-xylose depletion during mixotrophic batch processes, and only D-2,3-butanediol formation continued [33]. This sustained metabolic activity suggests that *C. autoethanogenum* LAbrini is more energetically resilient than the wild-type strain, supporting the increased robustness described by Ingelman et al. [43].

#### 3.2.2. Mixotrophic Batch Processes of *C. autoethanogenum* LAbrini with D-Fructose, D-Xylose, and L-Arabinose

**Figure 3:**
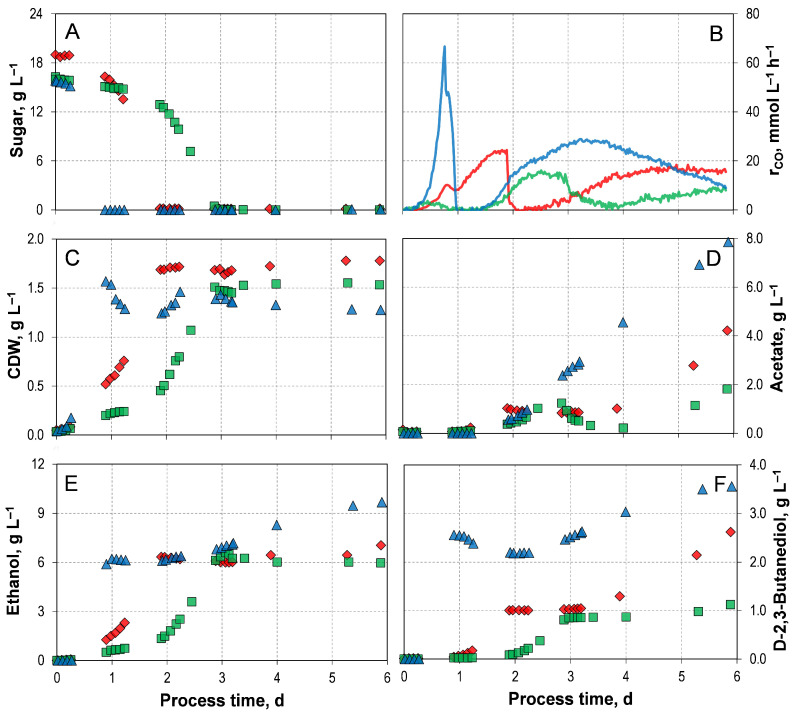
Mixotrophic batch processes of *C. autoethanogenum* LAbrini at varying initial sugar sources (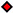 D-Fructose, 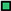 D-Xylose, and 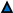 L-Arabinose) using autotrophic pre-cultures in stirred-tank bioreactors with continuous gassing (390 mbar N_2_, 300 mbar CO, 220 mbar H_2_, and 90 mbar CO_2_). (F_gas_ = 5 NL h^−1^, 1.0 bar_abs_, 37 °C, pH 6.0, and P V^−1^ = 15.1 W L^−1^). (**A**) Sugar concentration; (**B**) Carbon monoxide uptake rate; (**C**) Cell dry weight concentration (CDW); (**D**) Acetate concentration; (**E**) Ethanol concentration; (**F**) D-2,3-butanediol concentration.

Mixotrophic growth and product formation of *C. autoethanogenum* LAbrini, using either initial concentrations of 16.3 g L^−1^ D-xylose or 15.9 g L^−1^ L-arabinose, were also investigated employing the autotrophic pre-culture preparation in comparison to the mixotrophic growth process with D-fructose (Figure 3). The corresponding process performance data are summarized in Table 3.

All three mixotrophic batch processes were capable of metabolizing the supplied sugar and CO simultaneously (Figure 3A,B). However, the mixotrophic batch process involving D-xylose displayed a distinct five-phase profile of carbon uptake. Firstly, cells consumed only carbon through the WLP, followed by a phase of heterotrophic carbon utilization. This then transitioned into a mixotrophic phase. After the complete depletion of D-xylose, there was a brief decline in CO uptake, followed by a final phase of steady CO consumption. The total CO consumption of 747.10 mmol C L^−1^ is not significantly higher than in the autotrophic reference batch process.

The striking metabolic utilization of CO, in combination with D-xylose, results in significantly different product formation compared to those observed with D-fructose (Figure 3C–F). It takes three days for D-xylose to be consumed entirely, which is concomitant with the attainment of the maximum biomass concentration. Thereafter, the CDW concentration remained stable until the end of the process, similarly to the processes with D-fructose. However, the formation of the products did not start simultaneously. Ethanol was produced first, then acetate and D-2,3-butanediol. Interestingly, a temporary decrease in acetate concentration was observed. Additionally, a consistent drop in redox potential was detected before the acetate decreased in this process (Supporting Information; Figure S6). The same effect was observed in mixotrophic batch processes with *C. autoethanogenum* JA1-1, also using D-xylose as a heterotrophic substrate [33]. However, a substantial boost in ethanol formation was observed from acetate. The synchronous decrease in redox potential indicates an accumulation of reduced ferredoxin [33], suggesting a reduction through a ferredoxin-dependent aldehyde:ferredoxin oxidoreductase [20]. With *C. autoethanogenum* LAbrini, the reduction of ethanol was only slightly noticeable. However, once CO becomes the only substrate, the formation of alcohols ceased, and only acetate production continued. This suggests that the available reduction equivalents are no longer being channelled into these reduced product pathways, but are instead primarily required to support cellular maintenance metabolism. With *C. autoethanogenum* JA1-1, the formation of D-2,3-butanediol continued until the process ended [33]. However, it should be noted that this is based on phenomenological observations, as no intracellular redox potential measurements were performed.

In carbon uptake, the mixotrophic process with L-arabinose exhibited a three-phasic profile, similar to the process with D-fructose (Figure 3A,B). Initially, both substrates were utilized. Then, upon sugar depletion, there was a brief decline and succumb in CO uptake. Finally, purely autotrophic CO consumption occurred. In the mixotrophic batch process with L-arabinose, the sugar source was consumed in less than a day; this is significantly faster than in the other two processes. Furthermore, the maximum CO uptake rate of 66.34 mmol L^−1^ h^−1^ and the total CO consumption of 2376.87 mmol L were the highest compared to all the shown mixotrophic processes. For example, CO uptake rate and total CO consumption were four times, and three times, respectively, higher than in the process with D-xylose.

Here, too, the maximum CDW concentration was reached with the complete depletion of L-arabinose (Figure 3C). As with the mixotrophic process with D-fructose, the formation of all products followed a biphasic pattern (Figure 3D–F). The final acetate concentrations reached 7.86 gL^−1^, while the final concentrations of ethanol and D-2,3-butanediol were 9.68 g L^−1^, and 3.56 g L^−1^, respectively. This represents an increase of 533% (acetate), 47% (ethanol), and 300% (D-2,3-butanediol), respectively, compared to the mixotropic process with D-xylose.

**Figure 4:**
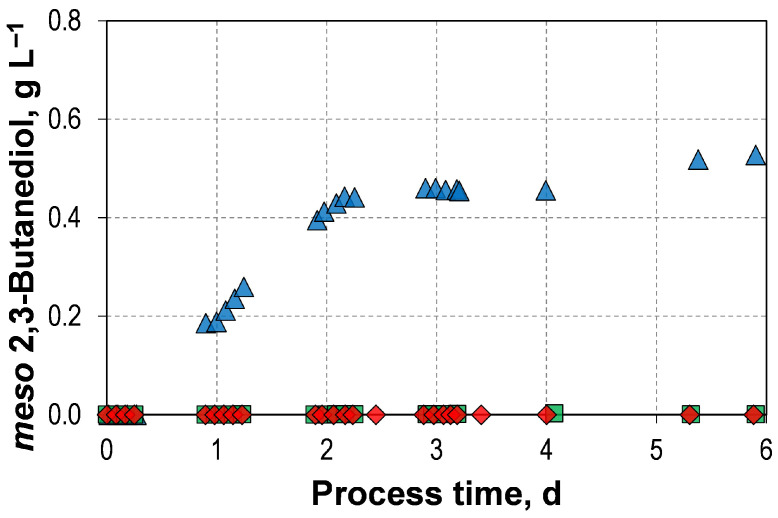
*meso*-2,3-butanediol formation at the mixotrophic batch process of *C. autoethanogenum* LAbrini with L-Arabinose (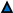) compared to the mixotrophic batch processes with D-Fructose (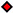) and D-Xylose (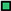) using autotrophic pre-cultures in stirred-tank bioreactors with continuous gassing (390 mbar N_2_, 300 mbar CO, 220 mbar H_2_, and 90 mbar CO_2_). (F_gas_ = 5 NL h^−1^, 1.0 bar_abs_, 37 °C, pH 6.0, and P V^−1^ = 15.1 W L^−1^).

Additionally, 0.53 g L^−1^ of *meso*-2,3-butanediol was also produced with L-arabinose (Figure 4). This stereoisomer is not present in the autotrophic reference process or in any of the mixotrophic batch processes involving D-fructose or D-xylose. However, it is known that a small amount of *meso*-2,3-butanediol can be formed in autotrophic cultivations with *C. autoethanogenum* JA1-1 [47]. Furthermore, our previous study using the wild-type strain found that *C. autoethanogenum* JA1-1 produced 1.08 g L^−1^ *meso*-2,3-butanediol in a mixotrophic batch processes with an initial concentration of 18.8 gL^−1^ L-arabinose [33]. The intracellular intermediate (R)-acetoin spontaneously racemizes via an enolate intermediate, meaning that some fraction of it is likely to be converted to (S)-aceton [48]. This can then be reduced to *meso*-2,3-butanediol [48]. Industrially, *meso*-2,3-butanediol is used as platform chemical for the production of thermoplastic polyurethanes [49,50]. While the other stereoisomers of 2,3-butanediol can also be used for this purpose, *meso*-2,3-butanediol is preferable due to the favourable spatial arrangement of its methyl side chains, which strengthens hydrogen bonding and therefore improves (thermo-)mechanical properties [49,51]. Furthermore, antibacterial and antiseptic properties have been observed in *meso*-2,3-butanediol-based polyurethanes [49].

**Figure 5:**
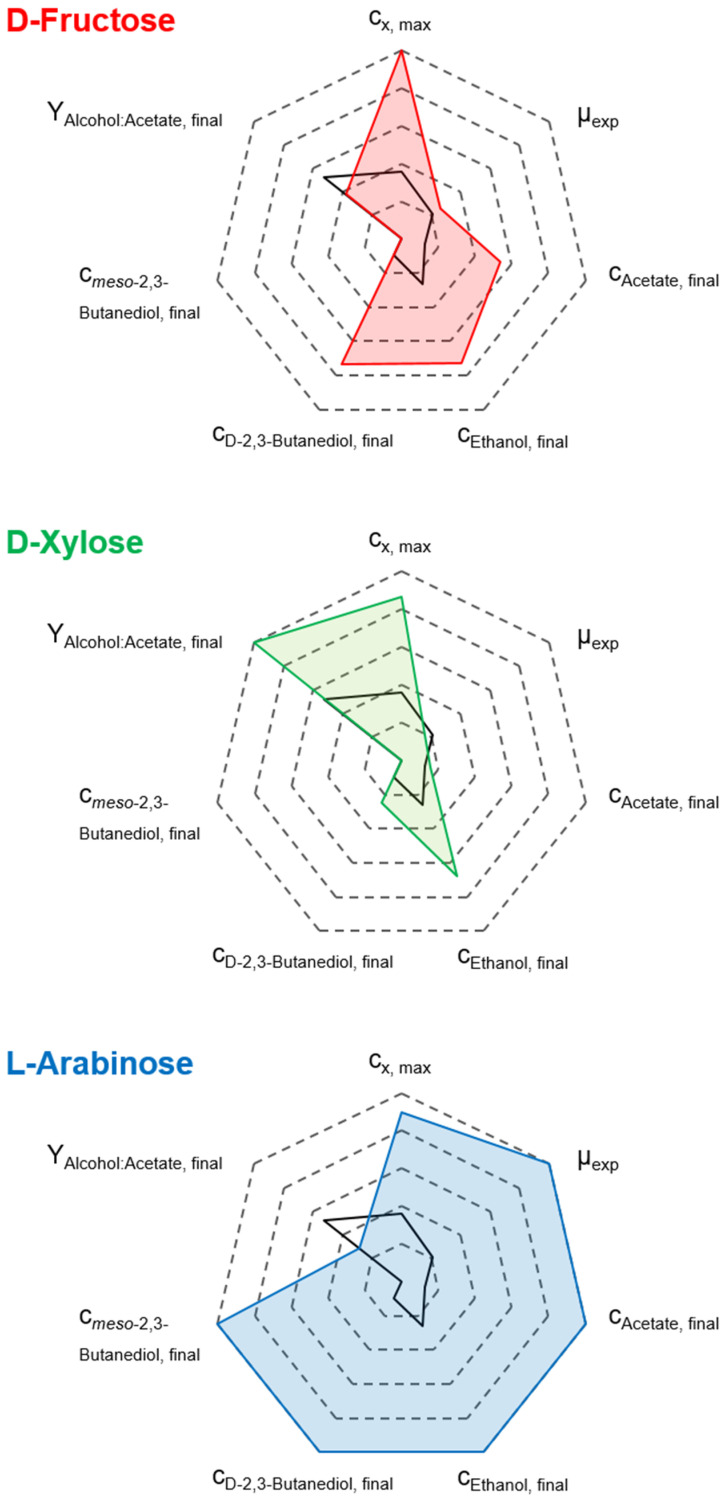
Comparison of c_x,max_, µ_exp_, c_p,final_, and alcohol-to-acetate ratio for the performed mixtrophic batch processes of *C. autoethanogenum* LAbrini with D-Fructose (red line), D-Xylose (green line), and L-Arabinose (blue line) compared to the autotrophic reference batch process (black line) in stirred-tank bioreactors with continuous gassing (390 mbar N_2_, 300 mbar CO, 220 mbar H_2_, and 90 mbar CO_2_). (F_gas_ = 5 NL h^−1^, 1.0 bar_abs_, 37 °C, pH 6.0, and P V^−1^ = 15.1 W L^−1^).

Overall, the final biomass concentration, exponential batch-specific growth rates, product concentrations, and alcohol-to-acetate ratios of the mixotrophic processes using D-fructose, D-xylose, and L-arabinose with the autotrophic pre-culture strategy are summarized in Figure 5. Further, the autotrophic reference batch process data was added for comparison.

Simultaneous utilization of CO and the respective supplied heterotrophic substrates was observed across all three mixotrophic processes. This proves that the PPP/EMP and WLP are operating in parallel. This resulted in higher biomass and elevated product formation compared to the autotrophic reference process. However, the metabolic patterns vary distinctly depending on the type of heterotrophic substrate supplied.

The mixotrophic batch process with D-fructose showed moderate final concentrations of alcohols. In contrast, the wild-type strain, which is unable to metabolize D-fructose and CO in parallel, showed only unfavorable acetate formation under comparable conditions [33], emphasizing the significant improvement achieved with *C. autoethanogenum* LAbrini. Reducing acetate to ethanol in the process with D-xylose resulted in the most favorable alcohol-to-acetate ratio of 6.04. Not only did the process using L-arabinose show the fastest sugar uptake, but it also produced the highest total alcohol concentration of 13.77 g L^−1^. Furthermore, L-arabinose was the only substrate that produced *meso*-2,3-butanediol under mixotrophic conditions.

## 4. Conclusions

Eliminating YE offers clear advantages for syngas fermentation, reducing costs and contamination risks. However, *C. autoethanogenum* LAbrini showed reduced growth and alcohol formation under YE-free conditions in controlled autotrophic batch processes with continuous gassing, demonstrating that this strain is still dependent on complex nutrients. This indicates a need for further strain development to achieve complete independence from complex nutrients.

Pre-culture preparation (autotrophic, mixotrophic, or heterotrophic) emerged as a critical step, causing substantial differences in growth, CO consumption, and product formation during mixotrophic syngas fermentation. Particularly promising was the mixotrophic batch process combined with a heterotrophic pre-culture, which enabled superior alcohol production. This finding has implications beyond *C. autoethanogenum* LAbrini, underscoring that pre-culture strategies must be considered as part of process design in CO-based syngas batch fermentation.

*C. autoethanogenum* LAbrini shows the ability to simultaneously metabolize CO and the supplied sugars. The co-utilization of D-fructose represents a notable improvement over the wild-type strain. Using an autotrophic pre-culture for a mixotrophic batch process with L-arabinose enhances CO consumption and alcohol formation, and solely enables the production of *meso*-2,3-butanediol. Additionally, L-arabinose can be obtained through the hydrolysis of lignocellulosic biomass, making it an attractive substrate for further mixotrophic syngas processes.

## Figures and Tables

**Table 1:** Process performance data of *C. autoethanogenum* LAbrini with 1 g L^−1^ yeast extract and without yeast extract (YE) in stirred-tank bioreactors with continuous gassing (artificial gas mixture of N_2_, CO, CO_2_, and H_2_ in a ratio of 39:30:22:9). (F_gas_ = 5 NL h^−1^, 37 °C, pH 6.0, and P V-1 = 15.1 W L^−1^) compared to process performance data of *C. autoethanogenum* JA1-1 (*) with 1 g L^−1^ yeast extract [33].

*Clostridium autoethanogenum*	JA1-1 (*) Min/Max	LAbrini Min/Max	LAbrini (Without YE)
Yeast extract, g L^−1^	1.0	1.0	-
µ_expt_, h^−1^	0.06	0.08–0.09	0.06
CDW, g L^−1^	0.49–0.54	0.63–0.65	0.44
c_Acetate,final_, g L^−1^	1.11–1.15	0.75–1.20	1.27
c_Ethanol,final_, g L^−1^	2.62–2.77	2.33–2.74	1.30
c_D-2,3-Butanediol,final_, g L^−1^	0.31–0.32	0.31–0.37	0.12
Ratio_Alcohol,final:Acetatefinal_, g g^−1^	2.64–2.68	2.20–4.14	1.12
Carbon in medium, mmol C L^−1^	9.94	9.94	0.90
Carbon in biomass, mmol C L^−1^	15.67–17.10	21.03–21.55	14.79
Carbon in products, mmol C L^−1^	147.15–160.19	144.61–148.17	101.83
CO consumption, mmol C L^−1^	626.31–652.06	598.16–632.37	359.09
CO_2_ production, mmol C L^−1^	400.96–438.80	443.41–445.97	212.00
CO cons./CO_2_ prod., -	1.49–1.56	1.34–1.41	1.69
CO consumption_max_, mmol L h^−1^	7.91–8.15	6.45–8.70	4.56
C-balance (recovery), %	90.66–91.10	96.44–99.82	91.29

**Table 2:** Process performance data of *C. autoethanogenum* LAbrini with D-Fructose using heterotrophic pre-cultures, autotrophic pre-cultures, and mixotrophic pre-cultures in stirred-tank bioreactors with continuous gassing (artificial gas mixture of N_2_, CO, CO_2_, and H_2_ in a ratio of 39:30:22:9). (F_gas_ = 5 NL h^−1^, 37 °C, pH 6.0, and P V^−1^ = 15.1 W L^−1^) compared to process performance data of *C. autoethanogenum* LAbrini with 1 g L yeast extract.

C_D-Fructose,initial_, g L^−1^	0 Min/Max	19.0	18.7	18.5
Pre-culture	autotrophic	autotrophic	mixotrophic	heterotrophic
µ_exp_, h^−1^	0.08–0.09	0.11	0.26	0.15
CDW, g L^−1^	0.63–0.65	1.78	1.94	1.66
c_Acetate,final_, g L^−1^	0.75–1.20	4.22	4.20	6.77
c_Ethanol,final_, g L^−1^	2.33–2.74	7.05	6.27	10.69
c_D-2,3-Butanediol,final_, g L^−1^	0.31–0.37	2.62	3.70	3.19
Ratio_Alcohol,final:Acetate,final_, g g^−1^	2.20–4.14	2.29	2.37	2.05
Carbon in medium, mmol C L^−1^	9.94	9.94	9.94	9.94
Carbon in biomass, mmol C L^−1^	21.03–21.55	59.32	64.60	55.35
Carbon in products, mmol C L^−1^	144.61–148.17	493.85	452.88	731.65
CO consumption, mmol C L^−1^	598.16–632.37	1699.65	2146.33	2644.26
CO_2_ production, mmol C L^−1^	443.41–445.97	1317.44	1723.32	2194.71
CO cons./CO_2_ prod., -	1.34–1.41	1.04	1.25	1.20
CO consumption_max_, mmol L h^−1^	6.45–8.70	26.02	23.88	32.64
C-balance (recovery), %	96.44–99.82	91.88	104.40	90.58

**Table 3:** Process performance data of *C. autoethanogenum* LAbrini with varying initial sugars (D-Fructose, D-Xylose, L-Arabinose) using autotrophic pre-cultures in stirred-tank bioreactors with continuous gassing (artificial gas mixture of N_2_, CO, CO_2_, and H_2_ in a ratio of 39:30:22:9). (F_gas_ = 5 NL h^−1^, 37 °C, pH 6.0, and P V^−1^ = 15.1 W L^−1^) compared to autotrophic process performance data of *C. autoethanogenum* LAbrini with 1 g L yeast extract.

c_sugar,initial_, g L^−1^	0 Min/Max	19.0 D-Fructose	16.3 D-Xylose	15.9 L-Arabinose
µ_exp_, h^−1^	0.08–0.09	0.11	0.07	0.42
CDW, g L^−1^	0.63–0.65	1.78	1.54	1.60
c_Acetate,final_, g L^−1^	0.75–1.20	4.22	1.24	7.86
c_Ethanol,final_, g L^−1^	2.33–2.74	7.05	6.59	9.68
c_D-2,3-Butanediol,final_, g L^−1^	0.31–0.37	2.62	0.89	3.56
c*_meso_*_2,3-Butanediol,final_, g L^−1^	0.00	0.00	0.00	0.53
Ratio_Alcohol,final:Acetatefinal_, g g^−1^	2.20–4.14	2.29	6.04	1.75
Carbon in medium, mmol C L^−1^	9.94	9.94	9.94	9.94
Carbon in biomass, mmol C L^−1^	21.03–21.55	59.32	51.76	52.30
Carbon in products, mmol C L^−1^	144.61–148.17	493.85	365.44	824.97
CO consumption, mmol C L^−1^	598.16–632.37	1699.65	747.10	2376.87
CO_2_ production, mmol C L^−1^	443.41–445.97	1317.44	819.54	1799.90
CO cons./CO_2_ prod., -	1.34–1.41	1.04	0.91	1.32
CO consumption_max_, mmol L h^−1^	6.45–8.70	26.02	16.11	66.34
C-balance (recovery), %	96.44–99.82	91.88	96.47	91.13

## Data Availability

The original contributions presented in the study are included in the article/Supplementary Materials; further inquiries can be directed to the corresponding author.

## Supplementary Materials

The following supporting information can be downloaded at https://www.mdpi.com/article/10.3390/microorganisms14010175/s1: Figure S1: (a) CO_2_ formation rate and (b) H_2_ uptake rate of autotrophic batch processes of *C. autoethanogenum* LAbrini with 1 g L^−1^ yeast extract () and without yeast extract () in stirred-tank bioreactors with continuous gassing (artificial gas mixture of N_2_, CO, CO_2_, and H_2_ in a ratio of 39:30:22:9). (F_gas_ = 5 NL h^−1^, 37 °C, pH 6.0, and P V^−1^ = 15.1 W L^−1^); Figure S2: Redox-potential of autotrophic batch processes of *C. autoethanogenum* LAbrini with 1 g L^−1^ yeast extract () and without yeast extract () in stirred-tank bioreactors with continuous gassing (artificial gas mixture of N_2_, CO, CO_2_, and H_2_ in a ratio of 39:30:22:9). (F_gas_ = 5 NL h^−1^, 37 °C, pH 6.0, and P V^−1^ = 15.1 W L^−1^); Figure S3: (a) CO_2_ formation rate and (b) H_2_ uptake rate of mixotrophic batch processes of *C. autoethanogenum* LAbrini with D-Fructose using autotrophic pre-cultures (), mixotrophic pre-cultures (), and heterotrophic pre-cultures () in stirred-tank bioreactors with continuous gassing (artificial gas mixture of N_2_, CO, CO_2_, and H_2_ in a ratio of 39:30:22:9). (F_gas_ = 5 NL h^−1^, 37 °C, pH 6.0, and P V^−1^ = 15.1 W L^−1^); Figure S4: Redox-potential of mixotrophic batch processes of C. autoethanogenum LAbrini with D-Fructose using autotrophic pre-cultures (), mixotrophic pre-cultures (), and heterotrophic pre-cultures () in stirred-tank bioreactors with continuous gassing (artificial gas mixture of N_2_, CO, CO_2_, and H_2_ in a ratio of 39:30:22:9). (Fgas = 5 NL h^−1^, 37 °C, pH 6.0, and P V^−1^ = 15.1 W L^−1^); Figure S5: (a) CO_2_ formation rate and (b) H_2_ uptake rate of mixotrophic batch processes of *C. autoethanogenum* LAbrini at varying initial sugar sources ( D-Fructose,  D-Xylose, and  L-Arabinose) using autotrophic pre-cultures in stirred-tank bioreactors with continuous gassing (artificial gas mixture of N_2_, CO, CO_2_, and H_2_ in a ratio of 39:30:22:9). (F_gas_ = 5 NL h^−1^, 37 °C, pH 6.0, and P V^−1^ = 15.1 W L^−1^). Figure S6: Redox-potential of mixotrophic batch processes of *C. autoethanogenum* LAbrini at varying initial sugar sources ( D-Fructose,  D-Xylose, and  L-Arabinose) using autotrophic pre-cultures in stirred-tank bioreactors with continuous gassing (artificial gas mixture of N_2_, CO, CO_2_, and H_2_ in a ratio of 39:30:22:9). (Fgas = 5 NL h^−1^, 37 °C, pH 6.0, and P V^−1^ = 15.1 W L^−1^). Table S1: Composition of the liquid cultivation medium (Doll et al., 2018 [41]) used for precultures in anaerobic shaken bottles and batch processes in stirred-tank bioreactors.
