# Supplementary material for: Autotrophic and Mixotrophic Batch Processes with Clostridium autoethanogenum LAbrini in Stirred Tank Bioreactors with Continuous Gassing
Source: Microorganisms. 2026 Jan 13;14(1):175. doi: 10.3390/microorganisms14010175 (PMC12844154; doi:10.3390/microorganisms14010175)
Supplement: Supplementary file 1 [file microorganisms-14-00175-s001.zip › microorganisms-4069435-supplementary.pdf]

microorganisms

MDPI

Supporting information

Table S1: Composition of the liquid cultivation medium (Doll et al., 2018 [41]) used for precultures in anaerobic shaken bottles and batch processes in stirred-tank bioreactors.

Component	Formula	Concentration in Stock solution, g L^{-1}
**Mineral solution**		**33.3x**
Ammoniumchloride	NH_4Cl	100
Natrium chloride	$NaCl$	80
Potassium chloride	KCl	10
Potassium dihydrogen phosphate	KH_2PO_4	10
Magnesium sulfate	$MgSO_4$	20
Calcium chloride	$CaCl_2$	4
**Vitamin solution**		**100x**
Pyridoxine	$C_8H_{11}NO_3$	0.01
Thiamine	$C_{12}H_{17}ClN_4OS$	0.005
Riboflavine	$C_{17}H_{20}N_4O_6$	0.005
Calcium pantothenate	$Ca(C_9H_{16}NO_5)_2$	0.005
Liponic acid	$C_8H_{14}O_2S_2$	0.005
Para amino benzoic acid	$C_7H_7NO_2$	0.005
Nicotinic acid	$C_6H_5NO_2$	0.005
Vitamin B12	$C_{72}H_{100}CoN_{18}O_{17}P$	0.005
D-biotine	$C_{10}H_{16}N_2O_3S$	0.002
Folic acid	$C_{19}H_{19}N_7O_6$	0.002
2 mercapto ethane sulfonic acid	$C_2H_6O_3S_2$	0.02
**Trace element solution**		**100x**
Nitrilotriacetic acid	$C_6H_9NO_6$	2.00
Mangan sulfate	$MnSO_4$	1.00
Ammonium iron sulfate	$NH_4Fe(SO_4)_2$	0.80
Cobalt chloride	$CoCl_2$	0.20
Zinc sulfate	$ZnSO_4$	0.20
Copper chloride	$CuCl_2$	0.02
Nickel chloride	$NiCl_2$	0.02
Sodium molybdate	Na_2MoO_4	0.02
Sodium selenate	Na_2SeO_4	0.02
Sodium wolframate	Na_2WO_4	0.02
		**Medium concentration**
**Yeast extract**		1.0 g L^{-1}
**Cysteine hydrochloride**	$C_3H_7NO_2S\ HCl$	0.4 g L^{-1}
**Morpholino ethane sulfonic acid**[a)]	$C_6H_{13}NO_4S$	15.0 g L^{-1}

a) only used for autotrophic and heterotrophic preculture in anaerobic shaken bottles

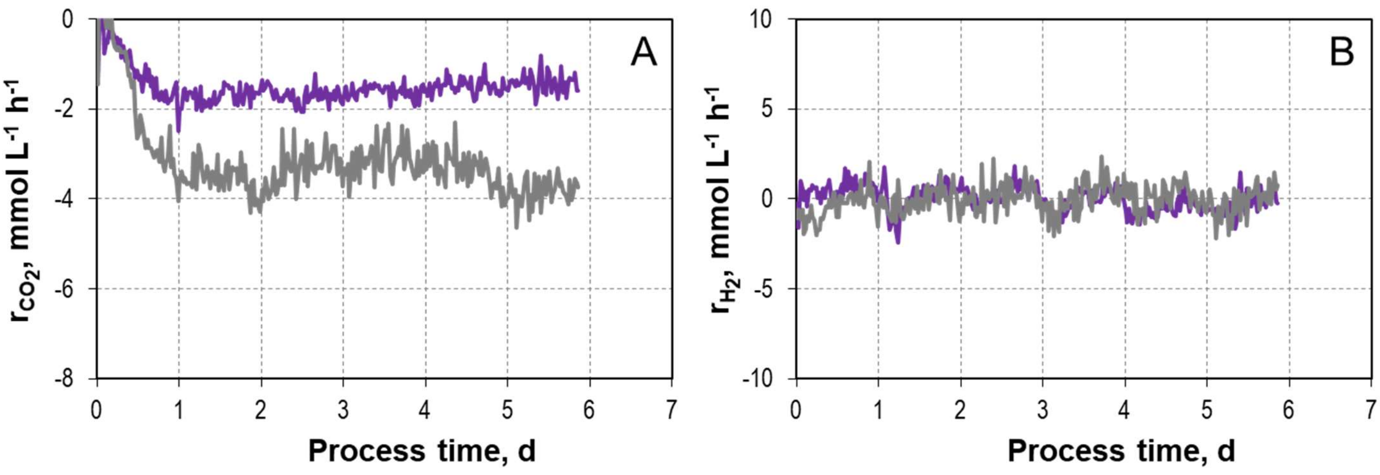

Figure S1: (a) CO_2 formation rate and (b) H_2 uptake rate of autotrophic batch processes of *C. autoethanogenum* LAbrini with 1 g L^{-1} yeast extract (○) and without yeast extract (○) in stirred-tank bioreactors with continuous gassing (artificial gas mixture of N_2, CO, CO_2, and H_2 in a ratio of 39:30:22:9). (F_{gas} = 5 NL h^{-1}, 37°C, pH 6.0, and P V^{-1} = 15.1W L^{-1}).

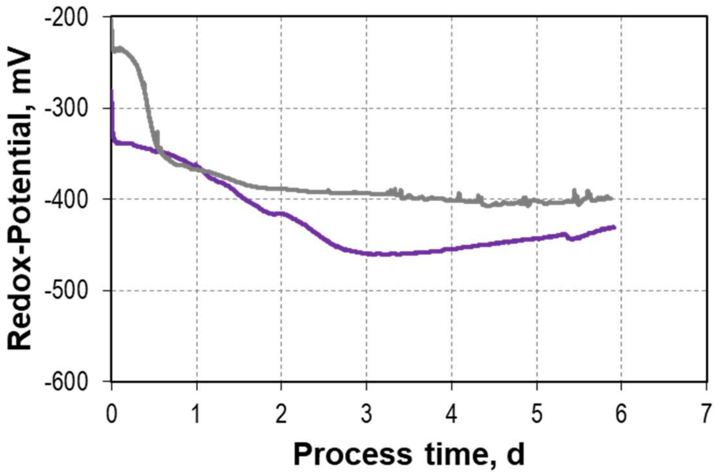

Figure S2: Redox-potential of autotrophic batch processes of *C. autoethanogenum* LAbrini with 1 g L^{-1} yeast extract (○) and without yeast extract (○) in stirred-tank bioreactors with continuous gassing (artificial gas mixture of N_2, CO, CO_2, and H_2 in a ratio of 39:30:22:9). (F_{gas} = 5 NL h^{-1}, 37°C, pH 6.0, and P V^{-1} = 15.1W L^{-1}).

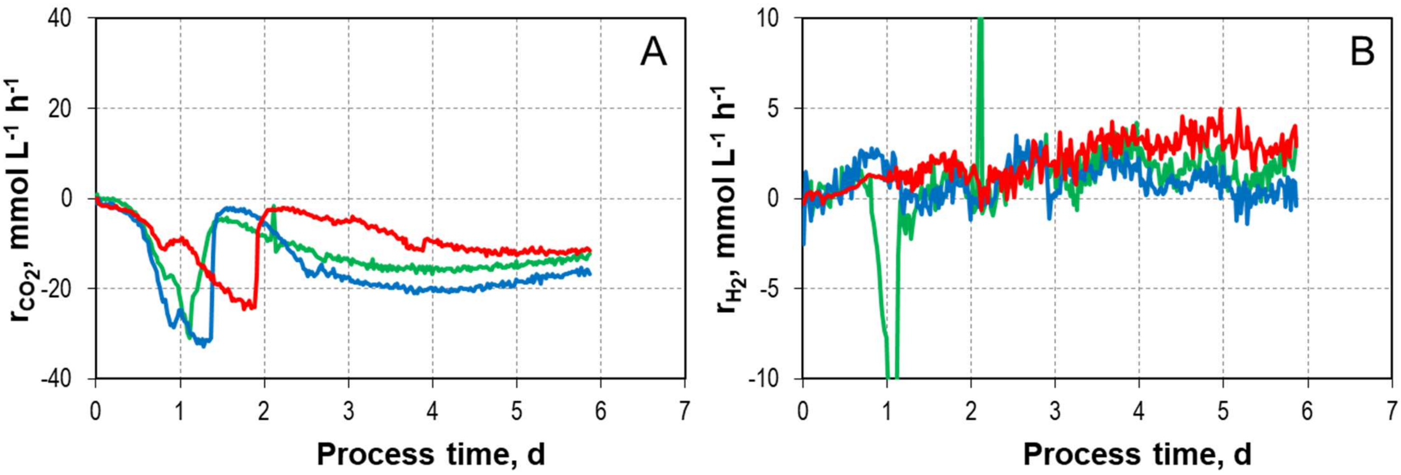

Figure S3: (a) CO_2 formation rate and (b) H_2 uptake rate of mixotrophic batch processes of *C. autoethanogenum* LAbrini with D-Fructose using autotrophic pre-cultures (◆), mixotrophic pre-cultures (◆), and heterotrophic pre-cultures (◆) in stirred-tank bioreactors with continuous gassing (artificial gas mixture of N_2, CO, CO_2, and H_2 in a ratio of 39:30:22:9). (F_{gas} = 5 NL h^{-1}, 37°C, pH 6.0, and P V^{-1} = 15.1W L^{-1}).

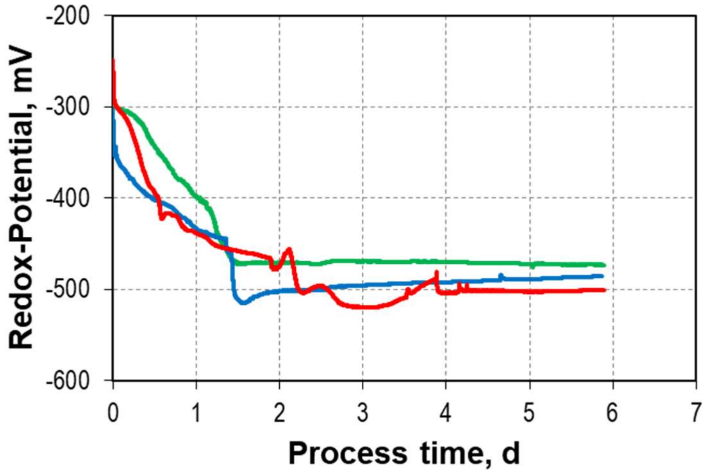

Figure S4: Redox-potential of mixotrophic batch processes of C. autoethanogenum LAbrini with D-Fructose using autotrophic pre-cultures (◆), mixotrophic pre-cultures (◆), and heterotrophic pre-cultures (◆) in stirred-tank bioreactors with continuous gassing (artificial gas mixture of N_2, CO, CO_2, and H_2 in a ratio of 39:30:22:9). (Fgas = 5 NL h^{-1}, 37°C, pH 6.0, and P V^{-1} = 15.1W L^{-1}).

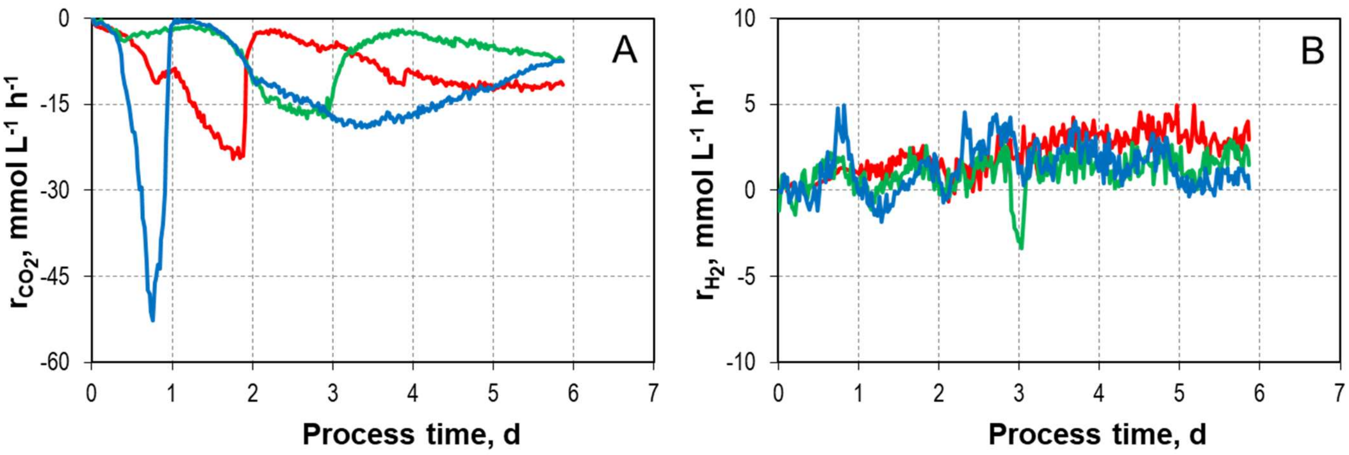

Figure S5: (a) CO_2 formation rate and (b) H_2 uptake rate of mixotrophic batch processes of *C. autoethanogenum* LAbrini at varying initial sugar sources (D-Fructose, D-Xylose, and L-Arabinose) using autotrophic pre-cultures in stirred-tank bioreactors with continuous gassing (artificial gas mixture of N_2, CO, CO_2, and H_2 in a ratio of 39:30:22:9). (F_{gas} = 5 NL h^{-1}, 37°C, pH 6.0, and P V^{-1} = 15.1W L^{-1}).

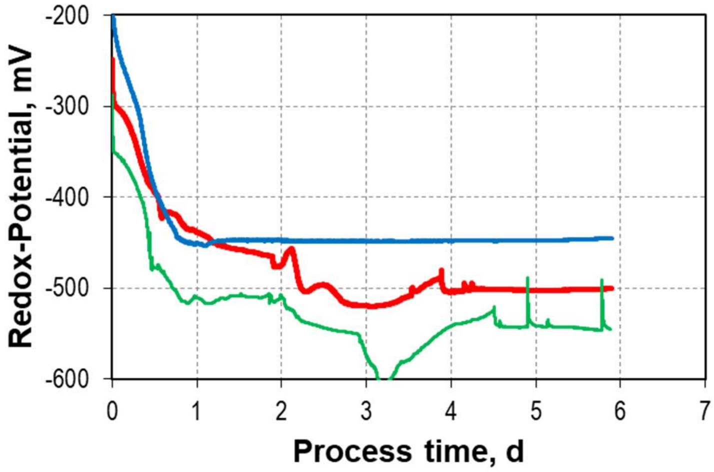

Figure S6: Redox-potential of mixotrophic batch processes of *C. autoethanogenum* LAbrini at varying initial sugar sources (D-Fructose, D-Xylose, and L-Arabinose) using autotrophic pre-cultures in stirred-tank bioreactors with continuous gassing (artificial gas mixture of N_2, CO, CO_2, and H_2 in a ratio of 39:30:22:9). (Fgas = 5 NL h^{-1}, 37°C, pH 6.0, and P V^{-1} = 15.1W L^{-1}).
